# Hypnosis-induced modulation of corticospinal excitability during motor imagery

**DOI:** 10.1038/s41598-020-74020-0

**Published:** 2020-10-09

**Authors:** Paola Cesari, Michele Modenese, Sara Benedetti, Mehran Emadi Andani, Mirta Fiorio

**Affiliations:** Department of Neurosciences, Biomedicine and Movement Sciences, University of Verona, Via Casorati, 43, 37131 Verona, Italy

**Keywords:** Neuroscience, Psychology, Human behaviour

## Abstract

Hypnosis can be considered an altered state of consciousness in which individuals produce movements under suggestion without apparent voluntary control. Despite its application in contexts implying motor control, evidence for the neurophysiological mechanisms underlying hypnosis is scarce. Inter-individual differences in hypnotic susceptibility suggest that sensorimotor strategies may manifest in a hypnotic state. We tested by means of transcranial magnetic stimulation applied over the primary motor cortex whether motor system activation during a motor imagery task differs in the awake and in the hypnotic state. To capture individual differences, 30 healthy volunteers were classified as high or low hypnotizable (Highs and Lows) according to ad-hoc validated scales measuring hypnotic susceptibility and personality questionnaires. Corticospinal activation during motor imagery in the hypnotic state was greater in the Highs than the Lows. Intrinsic motivation in task performance and level of persuasion modulated corticospinal activation in the Highs. Corticospinal system activation under hypnosis may have practical implications that merit research in areas where hypnosis can be applied to improve motor performance, such as loss of motor abilities and sports.

## Introduction

There is no clear consensus on whether hypnosis is an altered state of consciousness or simply a psychological state that emerges from the relationship between practitioner and patient^1^. Nevertheless, hypnosis is increasingly used for curing pain, depression, phobia and psychiatric disorders in clinical settings^2,3^ and to enhance attention and concentration on specific motor skills in sports^4^. Traditionally, research has focused on changes in cognitive components (particularly attentional) under the influence of hypnosis^5^, while less interest has been devoted to control of body movement. Pioneering studies suggested that hypnosis can induce a dissociation between the physical aspects of movement execution and the intention to move^1^. Only recently, however, has the question been more clearly formulated and experiments designed to determine whether the pre-planning and the intentionality of performing an action differ when measured under a hypnotic or in an awake state. Neural processes, for example, were measured in individuals asked to feel a limb paralyzed: in one group the suggestion was induced by an hypnotizer and in the other it was self-induced^6,7^.

Findings from imaging studies (fMRI, PET) and EEG recordings have been inconsistent and the brain mechanisms of hypnosis remain poorly understood^2^. It has been shown that voluntary movements in hypnotized individuals are accompanied by normal patterns of activation in motor and premotor areas of the brain^8^. Haggard et al.^6^ found that under hypnosis the mechanism of action control was not altered nor was the conscious experience of the action or the ability to make temporal judgments about actions. In general, they found that individuals planed their actions in a similar way irrespective of whether under hypnosis or in the awake state. A caveat to interpretation of their results is that only highly hypnotizable individuals were recruited for their study.

One of the critical aspects when dealing with hypnosis is the range of individual variability in the sensitivity of being hypnotized^9^. Sensitivity is evaluated by means of psychological multidimensional scales that include sensitivity for imagery, expectancy, attention/absorption and motivation^9,10^. For instance, individuals with elevated kinesthetic motor imagery are apt to be sensitive to hypnosis^3^. The hypothesis is that hypnosis mediates motor control by enhancing self-imagery and internal representation^11,12^: during hypnosis individuals are asked to engage in strategies that involve proprioception of movements when no actual movement occurs. Differences in behavioral^13,14^ and in neurophysiological parameters^15–17^ between highly- and low-hypnotizable individuals after receiving specific suggestions have been found.

The aim of the present study was to combine psychological and neuronal lines of research to better understand the effect of hypnosis on the control of actions. A professional hypnotizer (M.M.) categorized a pre-selected sample of 30 healthy volunteers by their level of sensitivity to hypnosis based on Stanford Hypnotic Susceptibility Scale scores^18,19^. Transcranial magnetic stimulation (TMS) was delivered to the left motor cortex while they imagined pressing their right thumb against their right index finger, and the amplitude of the motor evoked potentials (MEP) was recorded from two hand muscles. After completing the motor imagery task, they performed the actual action with the same two digits and the force was measured with a dynamometer. The experiment was performed in two different experimental conditions: the hypnotic and the awake state. With this experimental design we wanted to: (1) understand whether the motor system is differently activated when an individual is asked to imagine executing an action in the awake or the hypnotic state; (2) determine whether differences in motor system activation between the awake and the hypnotic state correspond to differences in force production; (3) investigate whether the level of individual sensitivity to hypnosis selectively modulates motor system activation in the awake and the hypnotic state.

## Methods

### Participant recruitment and hypnosis screening

Thirty health adults (13 females) were recruited from the student population of the University of Verona. Participation was on a voluntary basis and credits for internship were assigned as remuneration. The mean age was 22.33 ± 1.33 years (± standard deviation [SD]), all were right-hand dominant^20^, naive to the experiment, and had never experienced hypnosis.

Participants were screened for their degree of hypnotizability by a professional hypnotist (M.M.) in group sessions (6 participants each) during which he induced a hypnotic state and evaluated the ease with which a participant entered hypnosis. This was determined by following ad-hoc standardized maneuvers of the Hypnosis Stanford Hypnotic Susceptibility Scale procedure (SHSS)^19^ to evaluate the participant’s hypnotic susceptibility and the amount of time needed to enter the hypnotic state.

The psychologist (M.M.) followed each participant from the beginning to the end of the experimental procedure; he also instructed the participant on the imagery task and monitored the mental state of each participant. The SHSS score range is from 12 to 0. Participants with high hypnotic suggestibility (Highs) scored between 10 and 12 and those with low suggestibility (Lows) between 0 and 4. Since none of the Lows scored 0, we assumed that a certain level of suggestibility to hypnosis was present among all participants. The test involves the participant in 12 different situations guided by the hypnotist, plus an additional one in which the hypnotist delivers an individual posthypnotic suggestion. Each situation is evaluated by the hypnotist and scored 1 point. In this sample, the Lows were able to pass the first four situations but not the next ones. For instance, the Lows were able to experience the “arm lowering” situation (the upper arms are held out in front of the body and then lowered on command from the hypnotist) but not the “arm rigidity” and “amnesia” and no post-hypnotic command was given, but an inductive trace of concentration and attentional focus on the task.

The participants were comfortably seated in a large, quite room in such a way that the hypnotist could easily approach them. They were asked to relax and follow the hypnotist’s words as he guided them into a hypnotic state. He assessed the level of hypnotic susceptibility of each participant according to typical signs (e.g., head falling, eye closure, hand lowering, arm immobilization) that indicate response to hypnotic induction, as described in the SHSS^19^.

The participants were categorized according to their level of hypnotizability^21^. In the initial screening of the original sample of 30 individuals, 18 were categorized as highly-hypnotizable (Highs, 6 women, mean age 21.8 ± 1.75; SHSS score 10–12) and 11 as low-hypnotizable (Lows, 6 women, mean age 23.56 ± 2.92; SHSS score 1–4); one (female) scored 7 on the SHSS and so was excluded from the study. For the remaining participants, the hypnotist gave a posthypnotic suggestion, which was useful for quickly inducing the hypnotic state while testing the participants in the experimental session with TMS (see next section for details). Informed consent was obtained from all participants before starting the study. The study was approved by the departmental Committee for Approval of Research, University of Verona, and conducted according to the principles expressed in the Declaration of Helsinki.

### Neurophysiological measurement with transcranial magnetic stimulation (TMS)^22^

The participants were assigned to a single TMS session in which they were tested in the hypnotic and in the awake state following a counterbalanced order across participants to exclude learning and compensatory effects. Surface EMG was recorded from the motor point of the first dorsal interosseous (FDI) and abductor digiti minimi (ADM) muscles of the right hand, with bipolar self-adhesive Ag–AgCl electrodes (1.5 × 2.5 cm) in a belly-tendon montage. The ground electrode was attached to the wrist. EMG signals were band-pass filtered (20 Hz–2.5 kHz; plus 50 Hz notch) (D360, Digitimer, UK), amplified at a gain of 1000 (Digitimer), digitized at 5 kHz with laboratory interface (Cambridge Electronic Design 1401, UK) recorded by Spike 2 (version 6, Cambridge Electronic Design) and then analyzed off-line. A figure-of-eight coil (outer diameter of each wing 110 mm) was used to apply a biphasic single TMS pulse (STM 9000 magnetic stimulator, Ates-EBNeuro, Italy). The coil was mounted on an articulated arm and positioned on the left side and tangentially to the skull at a 45° angle to the sagittal plane^23^. The FDI optimal scalp position was identified by moving the coil in small steps laterally to the vertex in the left hemisphere and then delivering TMS pulses at constant intensity until stable and maximal MEPs were evoked in the relaxed FDI muscle. The resting motor threshold (rMT) was defined as the lowest stimulus intensity able to evoke MEPs with amplitude of at least 50 µV in at least five out of ten trials in the FDI muscle. During the experiment, the intensity of stimulation was set at 120% rMT^22^. MEP peak-to-peak amplitude was recorded from the two muscles (FDI and ADM). The pre-stimulation EMG level was evaluated by calculating the root mean square of the background EMG over 50 ms prior to MEP onset. Trials were removed in which the FDI MEP amplitude was lower than the mean background EMG. Moreover, all neurophysiological data were inspected to rule out outliers (i.e., values 2 × SD above or below the mean for each subject in each session).

### Procedure

Before starting the experiment, maximal voluntary contraction (MVC) was recorded by asking the participants to perform three trials in which they applied maximal pressure with the right thumb and index finger against a dynamometer (Hydraulic Hand Dynamometer SH5001, Saehan Corporation, South Korea). Participants learned to kinesthetically imagine the same pinch movement executed on the dynamometer during a brief training session in which they were asked to imagine the pinch pressure within a time interval between a vocal “go” and “stop” command by the experimenter. After each imagined movement, the participants had to say whether the time interval between the go and stop commands was long enough for them to imagine the movement. In the following training trials, the experimenter adapted the time interval according to the participants’ responses until duration of the time interval could be defined. In this way, we controlled the timing of the imagined movement for each participant. The SHSS allowed us also to define the participants’ kinesthetic imagery abilities. The SHSS includes suggestions to imagine and then practice different motor and proprioceptive experiences. For instance in item number 2, the participant is asked to hold his/her arms out in front of the body and to “imagine a force acting on your hands to push them apart, as though one hand were repelling the other. You are thinking of your hands being forced apart and they begin to move apart […]”^19^. While we could have applied the Movement Imagery Questionnaire (MIQ), we preferred to administer more detailed questionnaires that check for specific aspects related to individual suggestibility (like the MISS) and individual motivation and perception of adequacy in performing the task (like the IMI) (see next section for details).

**Figure 1:**
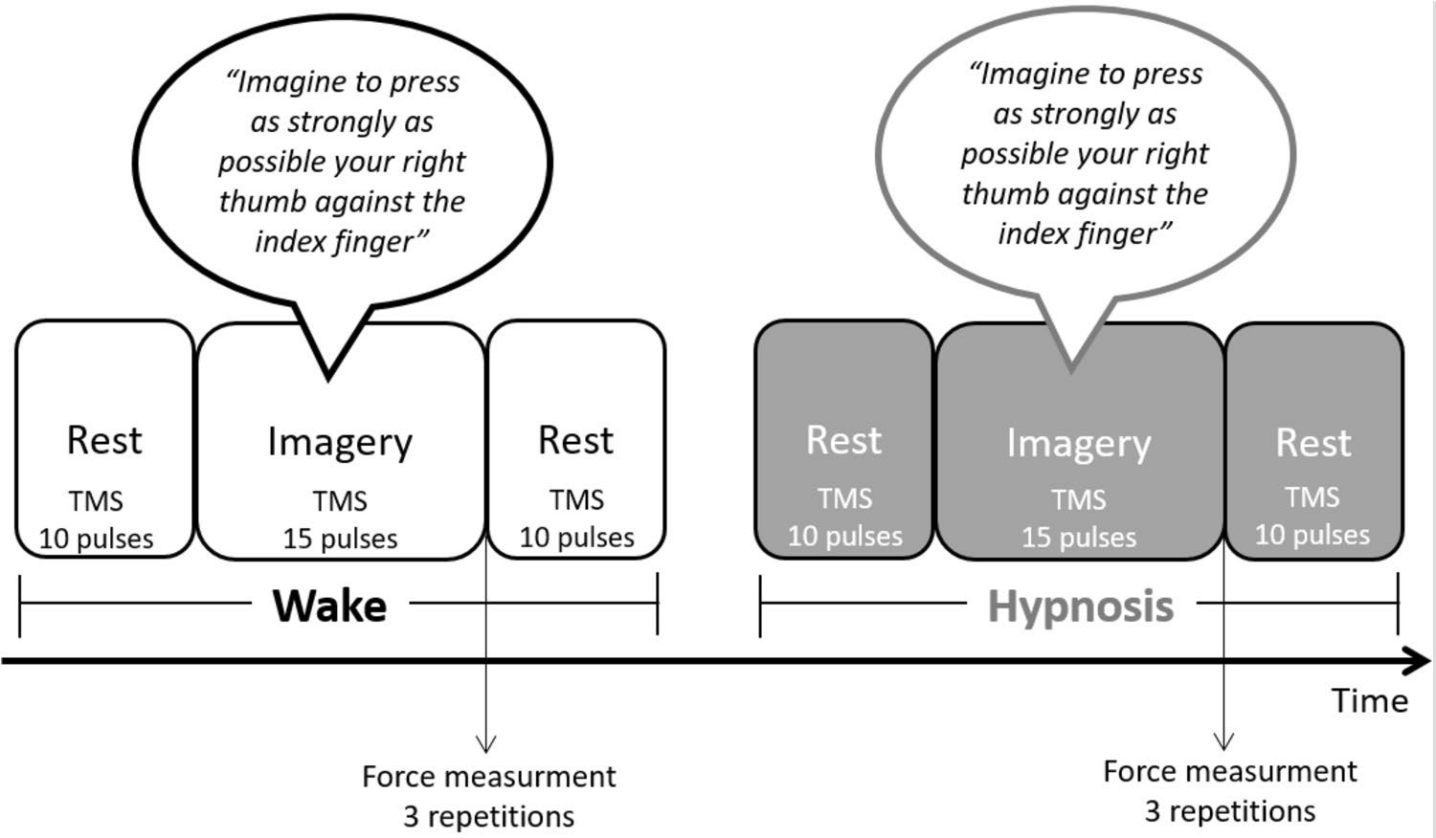
Experimental design. MEPs were recorded at rest and during imagination of a pinch movement of the thumb and index finger. Soon after the imagery task, participants were asked to apply actual force against a dynamometer. The same conditions were tested in the awake and the hypnotic state in a counterbalanced order across participants.

The MEP amplitude was recorded with the participants in two mental states (awake and hypnotic) and on two tasks (rest and motor imagery). The order of the awake and hypnotic states was counterbalanced across participants. For each mental state, we first recorded 10 MEPs at rest in order to have a measure of baseline excitability. The participants performed the imagination task (in the awake and the hypnotic state) and 15 MEP were recorded. For the motor imagery task, the participants started the imagined pinch movement after receiving a vocal “go” command from the experimenter. The TMS pulse was delivered manually within a time window that matched the duration of the imagined movement. Finally, 10 MEPs were recorded again at rest in order to have a measure of the final excitability level. At the end of each condition, the participants were asked to press the dynamometer three times and the force level was recorded. This allowed us to monitor changes in force production in the two mental states (awake vs. hypnotic). The duration of the hypnotic state was 20 min on average (Fig. 1).

### Dispositional traits

On completing the experimental procedure, the participants responded to two personality questionnaires that investigated the possible influence of dispositional traits in modulating the effects of hypnosis on corticospinal activation during imagination. Computerized questionnaires (E-prime, version 2.0) were used to facilitate data collection and processing. The Multidimensional Iowa Suggestibility Scale (MISS)^24^ is a 95-item questionnaire that measures social and psychological suggestibility defined as a tendency to accept messages from other individuals. It consists of five suggestibility subscales: consumer suggestibility, persuadability, physiological suggestibility, physiological reactivity, and peer conformity; the physiological suggestibility and reactivity subscales contain questions in which individuals are asked to imagine they are involved in specific situations and to perceive related sensations. The Intrinsic Motivation Inventory (IMI)^25,26^ is a 37-item multidimensional inventory that assesses subjective experience in relation to a target activity in laboratory experiments. It is subdivided in scales that assess interest/enjoyment, perceived competence and choice, effort perceived during a laboratory activity, value/usefulness of the activity, and pressure and tension felt during the activity. Three participants (2 Highs, 1 Low) did not complete the MISS questionnaire; 5 participants (3 Highs and 2 Lows) did not complete the IMI questionnaire.

### Data analysis

Repeated measure ANOVA of EMGrms showed no difference in muscle pre-activation (50 ms before applying the TMS trigger) between conditions (*p* = 0.411; Mean ± SE; WakeRest 3.14 ± 0.25 μV; WakeImagery 3.33 ± 0.24 μV; HypnoRest 3.11 ± 0.33 μV; HypnoImagery 3.42 ± 0.30 μV); 1.55% of the trials were removed because the FDI MEPs amplitude was lower than the mean background EMG. Neurophysiological measurements were checked to rule out outliers (i.e., values 2 × SD above or below the mean for each participant in each session); 3.57% of the trials were removed with this filter. The mean MEPs amplitude recorded in each mental state during rest and imagery was entered in the statistical analysis.

Due to the large inter-participant variability in MEPs amplitude, the data were transformed using z-scores, as described elsewhere^27^. Z-scores were calculated for each participant in each mental state (awake and hypnotic) following the formula:

$$Z \;score = \frac{{{\text{X}} - {\text{M}}}}{{{\text{SD}}}}$$

where X is the MEP amplitude of a single muscle in a specific condition, M and SD are, respectively, the mean and the standard deviation of the two muscles together (FDI and ADM) at rest. Z is the z-scores analyzed by repeated-measures ANOVA with State (awake and hypnotic), Task (rest and motor imagery), and Muscle (FDI and ADM) as within subjects factors and Group (Highs and Lows) as between-subjects factor. Post-hoc comparisons were performed with *t* tests for paired or independent samples; Bonferroni correction for multiple comparisons was applied where necessary. rmANOVA was performed to evaluate the amount of force applied in the two conditions for the two groups.

In order to evaluate whether corticospinal activation during the motor imagery task was further modulated by dispositional traits, the data collected via the two questionnaires (MISS and IMI) were correlated by Spearman’s coefficient of correlation with the MEP amplitude recorded during motor imagery from the FDI muscle (specifically involved in the imagined action) and from the ADM in the hypnotic and the awake state. Finally, *t* test for independent samples was used to compare the questionnaire responses between the two groups (Highs and Lows). Statistical significance was set at *p* < 0.050.

## Results

**Figure 2:**
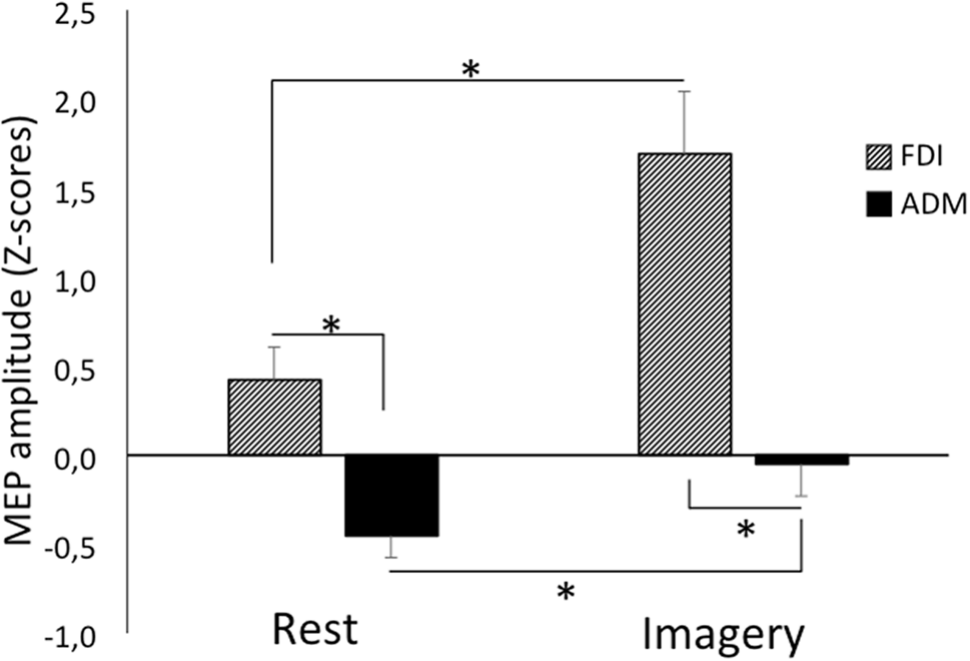
Mean ± standard error (SE) of the MEP amplitude (z-scores) recorded during rest and the motor imagery task from the two muscles: first dorsal interosseus (FDI) and abductor digiti minimi (ADM). Asterisks indicate significant comparisons (*p* < 0.05).

ANOVA showed significance for the main factors Task (F_(1,27)_ = 17.97, *p* < 0.001, ${\eta }_{p}^{2}=0.400$) and Muscle (F_(1,27)_ = 42.76, *p* < 0.001, ${\eta }_{p}^{2}=0.613$), indicating, respectively, higher motor system activation while the participants imagined they were producing force (mean ± standard error [SE] 0.78 ± 0.27) compared to rest (not imagining force production) (0.02 ± 0.15), and higher motor system activation for the FDI muscle (1.05 ± 0.27) than the ADM muscle (− 0.25 ± 0.16). The Task × Muscle interaction was significant (F_(1,27)_ = 23.16, *p* < 0.001, ${\eta }_{p}^{2}=0.462$). Post-hoc comparisons showed that both muscles were significantly more active during motor imagery compared to rest and that the FDI was more active than the ADM at both motor imagery and rest (for all comparisons, *p* < 0.001, Cohen’s d effect size: d > 1.83) (Fig. 2).

**Figure 3:**
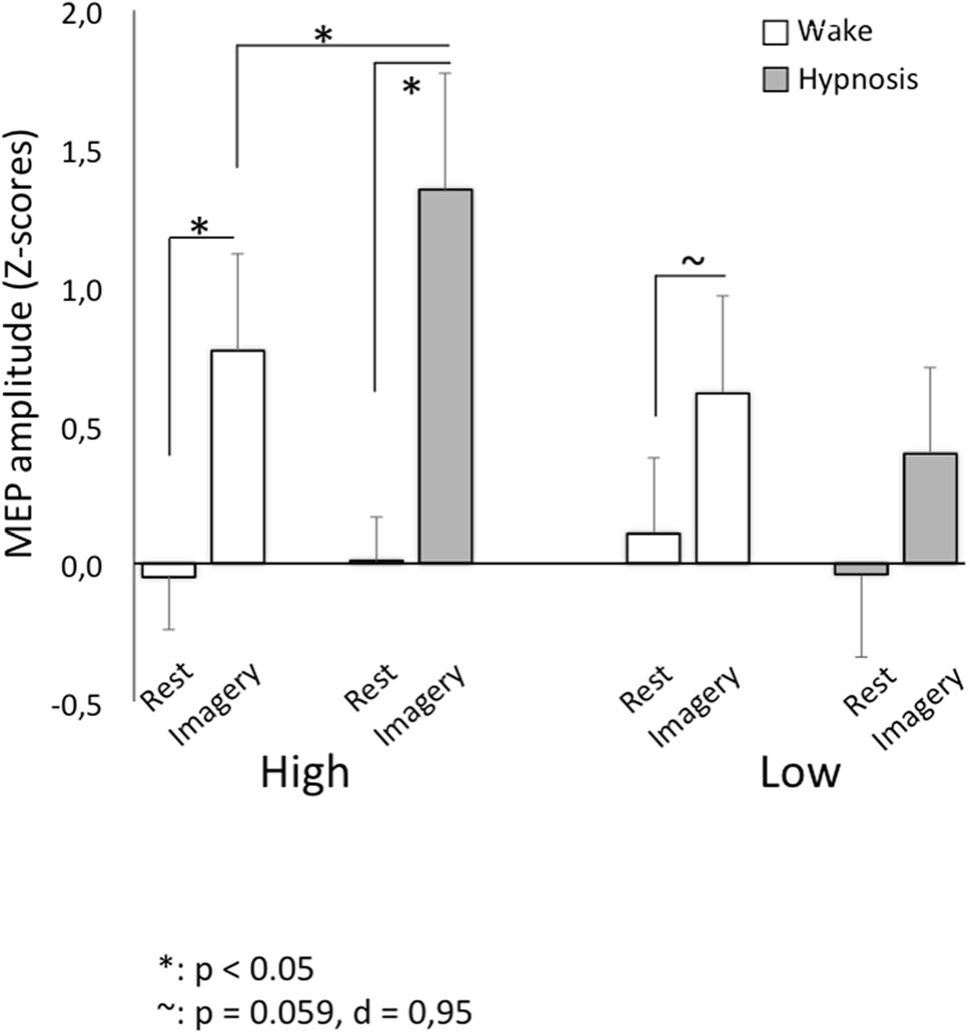
Mean ± standard error (SE) of the MEP amplitude (z-scores) recorded at rest and during the motor imagery task in the awake and the hypnotic state for the Highs and Lows. Asterisks indicate significant comparisons (*p* < 0.05).

The Group X State (F_(1,27)_ = 4.94, *p* = 0.035, ${\eta }_{p}^{2}$= 0.155) interaction showed higher activation during the hypnotic state than the awake state for the Highs (0.68 ± 0.28 and 0.36 ± 0.26, respectively; *p* = 0.010, d = 0.99) (Fig. 3). Finally, the triple interaction Task × State × Group was significant (F_(1,27)_ = 4.87, *p* = 0.036, ${\eta }_{p}^{2}$= 0.462). Post-hoc comparisons showed higher activation during the hypnotic state than the awake state the Highs (1.35 ± 0.42 and 0.77 ± 0.35, respectively; *p* = 0.002, d = 1.29) on the imagery task (Fig. 3). Furthermore, activation was higher during the imagery task than rest in the awake (imagery 0.77 ± 0.35, rest − 0.05 ± 0.19; *p* = 0.002, d = 1.29) and the hypnotic state for the Highs (imagery 1.35 ± 0.42, rest: 0.01 ± 0.16; *p* < 0.001, d = 1.59). In contrast, a trend for higher imagery activation compared to rest during the awake state was noted for the Lows (imagery 0.61 ± 0.35, rest 0.11 ± 0.28; *p* = 0.059, d = 0.95). No other effects or interactions were statistically significant (for all comparisons, *p* > 0.119).

To further support the results, we clustered the increment of the corticospinal excitability (Δ = MEPTask − MEPRest) by applying the k-means clustering method considering the two groups in the two mental states separately: Wake and Hypnosis. In the Hypnosis state, the results revealed that the odds of Δ in High group was estimated to be 4.19 times the odds of Delta in Low group (95% Confidence interval (95% CI) [2.30, 7.59]). Both odds ratio and 95% CI range are greater than one, indicating a good discrimination between the groups: higher corticospinal excitability for the High compared to the Low group. Moreover, the level of F1-score is 0.65 showing a good clustering result. In the Wake state, the results revealed that the odds of Δ in the High group was estimated to be 1.79 times the odds of Delta in the Low group (95% CI [0.80, 3.98]). In this case while the odds ratio is greater than one, the 95% CI values are less than one for the lower side and more than one for the higher side of the range, indicating a weak in discriminating between the High and Low groups. Furthermore, the level of F1-score is 0.51 showing a weak clustering result.

No significant difference in actual force production measurements between the awake and the hypnotic state or between the two groups was found (for all factors, *p* > 0.110). The force data for the two experimental conditions for the two groups were: hypnotic state—Highs 7.66 ± 4.23; Lows 8.68 ± 2.72; awake state—Highs 7.64 ± 3.51; Lows 7.68 ± 3.42.

Correlations between dispositional traits and amplitude of the FDI and ADM MEPs recorded during the motor imagery task were found only for the Highs. There was a positive correlation between the amplitude of the FDI MEPs and the IMI “choice” subscale scores in the awake and the hypnotic state (awake state rho = 0.690, *p* = 0.004; hypnotic state rho = 0.574, *p* = 0.025) (Supplementary Fig. S1A,B). A positive correlation was found also for the AMD but only in the hypnotic state (rho = 579, *p* = 0.024) (Fig. S1C,D). Conversely, a negative correlation was found between the amplitude of the FDI MEP and the IMI “pressure/tension” subscale scores, in the awake and the hypnotic state (awake state rho = − 0.541, *p* = 0.037; hypnotic state rho = − 0.637, *p* = 0.011) (Fig. S2A,B). There was a positive correlation between the amplitude of the FDI MEP and the IMI “effort” subscale score in the awake state (rho = − 0.586, *p* = 0.022) (Fig. S2C,D).

Correlations were significant only for the Highs in the awake and the hypnotic state between the FDI MEP amplitude and the MISS “persuasion” subscale scores (awake state rho = 0.534, *p* = 0.033; hypnotic state rho = 0.682, *p* = 0.004) (Fig. S3A,B). The scores also correlated with the ADM but only in the hypnotic state (rho = 0.528, *p* = 0.036) (Fig. S3C,D).

Comparison of dispositional traits between the two groups revealed higher IMI pressure/tension subscale scores (3.93 ± 1.23) for the Highs than the Lows (2.25 ± 0.83) (t_(21)_ = 3.45, *p* = 0.002) (Fig. S4). No other difference in personality trait between the two groups was found.

## Discussion

This experiment enabled us to better understand whether the motor system is differently activated when an individual is asked to imagine executing an action in the awake and the hypnotic state and whether activation differs between individuals who are more or less susceptible to hypnosis. The present findings provide evidence for the idea that imagining a movement in the hypnotic state modulates corticospinal system excitability depending on an individual’s sensitivity to hypnosis. In this sample of healthy volunteers, M1 activity during force production imagination was increased significantly in the hypnotic state compared to the awake state in participants categorized as Highs. In addition, correlations between the level of M1 activity and the level of intrinsic motivation in performing the task, along with a high level of persuadability for the suggestions delivered by the hypnotist, were noted only for the Highs. In contrast, no difference in MEP amplitude between rest and imagery in hypnosis (as noted instead in the awake state) was found for the Lows and no correlation with dispositional traits was observed. The difference in neurophysiological patterns suggests that corticospinal system activation during internal simulation of actions can be influenced by individual factors in the hypnotic and the awake state. This finding can be explained by taking into account the mechanisms underlying the hypnotic state.

Feed-forward control for action and top-down control mechanisms are fundamental for hypnotic phenomenology^6^ (e.g., the control of actions and postures and the ability to compensate for reduced or altered sensory availability and attention) based on attention/imagery/memory systems that generate an internal world congruent with reality^28,29^. Studies on hypnosis report that the physiological and behavioral effects of hypnotic suggestion are a function of an individual’s level of hypnotizability^9^. The connection between hypnotizability and motor control relies on the assumption that Highs and Lows may differ in many ways, including cognitive ability (e.g., voluntarily directed and sustained attention^30^) and action-based ability (e.g., control of action planning^10,21,31–33^). Our results show that being highly or low sensitive to hypnosis does not alter motor cortex activity during the resting state. In other words, when the participants were not engaged in a task, (e.g., in resting condition), the level of motor cortex activation did not change between the awake and the hypnotic state; instead when the participants were asked to internally simulate force production, the motor cortex activation patterns differed between the Highs and the Lows. For the Highs, more activation was observed in both imagery mental states but it was greater during the hypnotic state. Differently, motor cortex activity was slightly higher for the Lows during the imagination task in the awake condition, but significant activation was absent when the Lows were in the hypnotic state.

These findings suggest that the Highs experienced the imagery of actions more effectively than the Lows and that, through imagery, cortical activation was increased due to hypnotic suggestion. Indeed, previous studies have shown that the central programs for motor control seem to be better updated in Highs than in Lows^34,35^, and it is likely that a better control of actions relies also on the ability to quickly and more effectively incorporate a hypnotist’s suggestions.

In line with previous work relating the level of hypnosis susceptibility with cortical activity, we may speculate that the parietal cortex is a good candidate as a neural substrate of the modulation found in our study. Namely, besides being involved in the formation of body image in relation to external space, the parietal cortex works as a multimodal region for the control of force and posture^36^. Other brain structures that may possibly discern between Highs and Lows are the corpus callosum^35^, the locus coeruleus, and the frontal lobe^37,38^. In individuals highly susceptible to hypnosis, the corpus callosum has a larger anterior part that likely allows better inter-hemispheric transfer of information and executive processing; while the frontal lobe, the principal source of ascending and descending noradrenergic cerebral pathways, has gained interest in the field of hypnosis because it is involved in attentional mechanisms and is responsible for the level of alertness^39,40^.

Much of the research on hypnosis has been focused on inducing suggestions that alter perception, such as feeling an arm frozen or anesthetized^7,41^. Findings have revealed a consistent congruency between the suggestions delivered by the hypnotist and the physiological and behavioral reactions in highly hypnotically susceptible individuals. In previous studies, individuals under hypnosis were induced to imagine they perceived sensory states, whereas in our study the participants were asked to imagine they were performing actions. Neurophysiological^27^ and neuroimaging studies^42–47^ showed the existence of a motor resonant mechanism for action in the awake state in humans^48^. TMS studies demonstrated that the mere observation or imagination of an action induces a selective increase in MEPs recorded from the muscles that would be active if the observed or imagined actions were performed^27,49^. Here, we showed that in individuals highly or less sensitive to hypnotic suggestion there is a selective activation of the muscles that they imagined they had to contract to produce force^50^. Interestingly though, when the imagination was guided under hypnosis, the resonant mechanism was increased only in the Highs, which corroborates the hypothesis that hypnosis mediates the control of actions by enhancing self-imagery of internal representation^11,12^. This contrasts with the weak resonant mechanism observed in the Lows, particularly under the hypnotic state, and provides evidence for the lack of a kinesthetic internal representation^21^.

Our results are shared by Cojan et al.^7^ who showed that, differently from the controls, prefrontal and parietal cortices activity was increased in the Highs when asked to imagine and execute arm movements. Since these areas are involved in the executive control of action and attention, we suggest that hypnosis mediates the control of movements by internal representations that are triggered by self-imagery^7^. A future area of focus is to test the effect that motor imagery training under hypnosis can have on learning how to efficiently apply ideomotor strategies during actual performance^51^. In general, Highs are noted to gather sensorimotor information through imagination better and process it more effectively than Lows, in addition to greatercongruent cortical activation^50^. Nonetheless, greater corticospinal activation does not necessarily mean stronger force production: no force modulation was found when we compared force production in the awake and the hypnotic state or between the Highs and the Lows. This suggests that the differences in corticospinal activation were specific for the imagery task and do not influence the ability to perform the actual force production task^6, 7^. Future research will need to increase the number of trials and use a more sensitive and accurate instrument for collecting actual force data. For example, a 3D force cell would be optimal for measuring not just the grip force but also the force increment modulated in this task.

Analysis of dispositional traits revealed that corticospinal activation during imagination was further modulated by personality traits only for the Highs. For instance, the IMI subscale “perceived choice” scores positively correlated with corticospinal excitability, indicating that corticospinal system activation in the hypnotic and awake state was higher the Highs who felt freer in performing the task. Because the perceived choice subscale is a positive predictor of self-report measures of intrinsic motivation^25,26^, this finding suggests that individual differences in the level of intrinsic motivation could account for further variability in corticospinal system activation during the hypnotic and the awake state in individuals who are highly sensitive to hypnosis. The negative correlation for the Highs between corticospinal activation and the pressure/tension subscale is consistent with this line of reasoning, since it is designed to be a negative predictor of intrinsic motivation^25,26^. Intrinsic motivation in performing the task is further sustained by the positive significant correlation between the IMI subscale “effort” and corticospinal activation during the imagery task in the awake state, again, only for the Highs. The subscale “effort” investigates an individual’s engagement in a task at hand. Hence, this correlation indicates higher corticospinal activation for the Highs, who felt more engaged in the imagery task.

High hypnotic suggestibility facilitates attentional mechanisms and enhances visuo-proprioceptive multisensory integration^52^. Moreover, Highs are noted to present a well-developed multisensory integration besides a higher sense of embodiment^53^ and this may have boosted involvement in performing the task at hand. Finally, correlations between neural activity and MISS questionnaire responses were observed for the Highs. The MISS measures degree of suggestibility, a personality trait that reflects an individual’s general tendency to accept suggestions. In our sample, a positive correlation between corticospinal activation and the MISS subscale persuasion was noted for the Highs in the awake and the hypnotic state. In general, all significant correlations were present for the Highs and all involved the FDI as the target muscle. The IMI questionnaire responses (perceived choice; pressure/tension, and effort) indicate that the participants felt free and adequate in performing the task and highly motivated. The MISS questionnaire responses indicated, again only for the Highs, a general open attitude toward accepting suggestions.

We would like to mention several exclusion factors that may help to sustain the uniqueness of the hypnotic state. We can exclude the possibility that the participants were simply relaxed rather than hypnotized because, were such the case, we would expect to find smaller MEPs amplitudes compared to the resting condition^54^. Moreover, the EMGrms suggest that all participants presented the same level of muscle activity in the hypnotic and the awake state. Highs are known to present normally higher kinesthetic imagery^55^; nevertheless, while no additional commands to increase the imagination capacity were given during hypnosis, we cannot exclude the possibility that the Highs might have amplified their imagination and thus increased their MEPs. Finally, we can exclude any effect of learning and or fatigue, since the experimental protocol was counterbalanced between the participants.

Future studies involving larger samples are desirable to investigate the connections between susceptibility to hypnosis, intrinsic motivation, and corticospinal excitability. Furthermore, the use of the hypnotic state while individuals are engaged in a motor imagination task might help to improve our understanding of sensorimotor integration. In other words, the combined study of human behavior and related neuronal underpinnings in individuals under the influence of diverse “states of mind” might reveal basic mechanisms of perception–action coupling. Moreover, understanding the mechanisms responsible for the differences between Highs and Lows may help to define strategies that individuals can use to plan their actions under the influence of different mental states. For instance, the ability to experience by imagining sensory and motor information as true perception, even under the influence of different states of mind (awake and hypnosis), may indicate a good level of plasticity of the central motor programs in terms of flexibility in switching between internal reference systems, as sustained by several authors^56–58^.

One of the relevant aspects of the present experiment is that brain activity and force production were measured while the participants were under hypnosis or in the awaking state. It is important to note that much of the research on hypnosis evaluated subjects’ performance before and after the induction of hypnosis. Studying corticospinal system activation during hypnosis can have important future implications for conditions in which it is used to improve motor performance, as in loss of motor abilities and in sports. Moreover, we may speculate that the level of hypnotizability might be a useful index to predict individual reactions in many contexts. For instance, the ability of Highs to modulate their control of actions based on a change in mental state may encourage them to take advantage of treatment (e.g., for ageing or illness), with a better prognosis achieved through neuro-rehabilitation centered on imagery training. Also, Highs are particularly receptive to adapting to environments where sensory inputs are different from normal (e.g., space flight, extreme sports). Finally, we wish to conclude with the words of John Kihlstrom^59^ that well summarize the main results of this experiment: “[…] the hypnotizable brain, even when hypnotized, is just like any other brain - only better”.

## Supplementary information

Supplementary Legends.

Supplementary Figure S1.

Supplementary Figure S2.

Supplementary Figure S3.

Supplementary Figure S4.

## Data Availability

The data generated and analyzed during this study are available from the corresponding author on reasonable request.
