# Supplementary material for: Hypnosis-induced modulation of corticospinal excitability during motor imagery
Source: Sci Rep. 2020 Oct 9;10:16882. doi: 10.1038/s41598-020-74020-0 (PMC7547693; doi:10.1038/s41598-020-74020-0)
Supplement: Supplementary file 1 — Supplementary Legends. [file 41598_2020_74020_MOESM1_ESM.docx]

**Supplementary information**

**Hypnosis-induced modulation of corticospinal excitability during motor imagery.**

Paola Cesari*, Michele Modenese, Sara Benedetti, Mehran Emadi Andani, Mirta Fiorio

Department of Neurosciences, Biomedicine and Movement Sciences, University of Verona, Italy

***** paola.cesari@univr.it

**Legends to supplementary figures**

**Figure S1**. Correlations between scores at the IMI “perceived competence” subscale and MEP amplitude (expressed as Z-scores) obtained during motor imagery from the FDI (upper panels, A and B) and ADM (lower panels, C and D) muscle, in normal wake (left panels) and in hypnosis (right panels). Grey circles represent data of Highs and white squares represent data of Lows. Continuous lines represent significant correlations in the group of Highs.

**Figure S2**. Correlations between MEP amplitude (expressed as Z-scores) obtained during motor imagery from the FDI muscle and scores at the IMI “pressure/tension” subscale (upper panels, A and B) and the IMI “effort/importance” subscale (lower panels, C and D), in normal wake (left panels) and in hypnosis (right panels). Grey circles represent data of Highs and white squares represent data of Lows. Continuous lines represent significant correlations in the group of Highs.

**Figure S3**. Correlations between scores at the MISS “persuasion” subscale and MEP amplitude (expressed as Z-scores) obtained during motor imagery from the FDI (upper panels, A and B) and ADM (lower panels, C and D) muscle, in normal wake (left panels) and in hypnosis (right panels). Grey circles represent data of Highs and white squares represent data of Lows. Continuous lines represent significant correlations in the group of Highs.

**Figure S4**. Mean values and standard deviations of Highs (grey bars) and Lows (white bars) in the subscale of the IMI questionnaire. Highs had higher scores than Lows at the pressure/tension subscale. * p < 0.050.
