# Supplementary figures and images for: Hypnosis-induced modulation of corticospinal excitability during motor imagery
Source: Sci Rep. 2020 Oct 9;10:16882. doi: 10.1038/s41598-020-74020-0 (PMC7547693; doi:10.1038/s41598-020-74020-0)

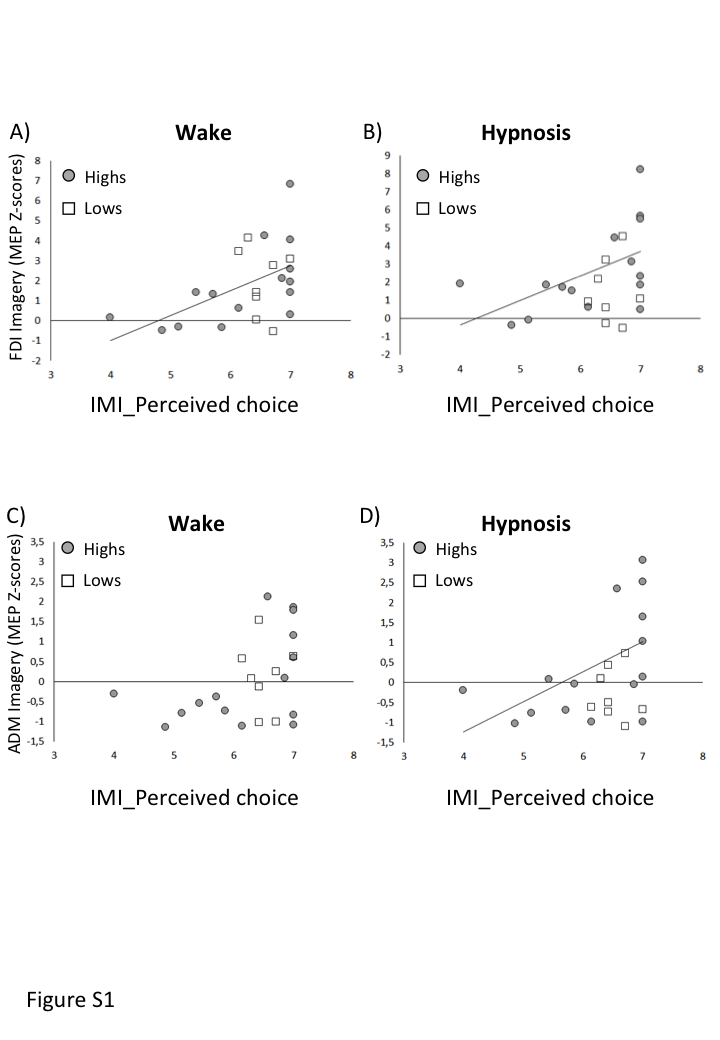

Supplement: Supplementary file 2 — Supplementary Figure S1. [file 41598_2020_74020_MOESM2_ESM.tiff]

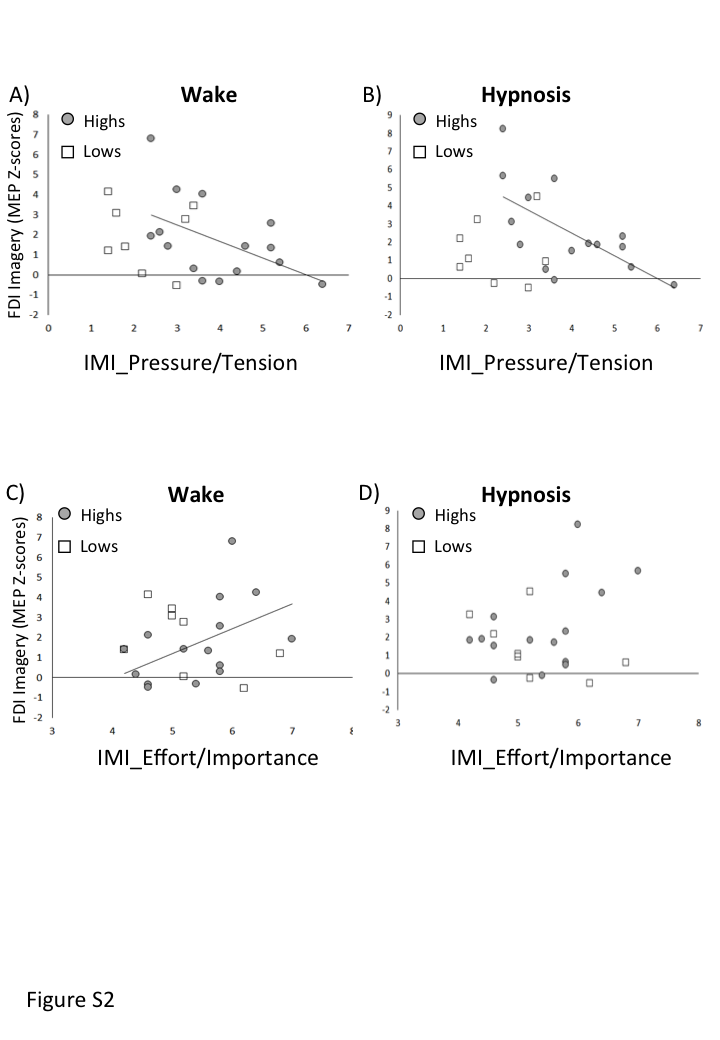

Supplement: Supplementary file 3 — Supplementary Figure S2. [file 41598_2020_74020_MOESM3_ESM.tiff]

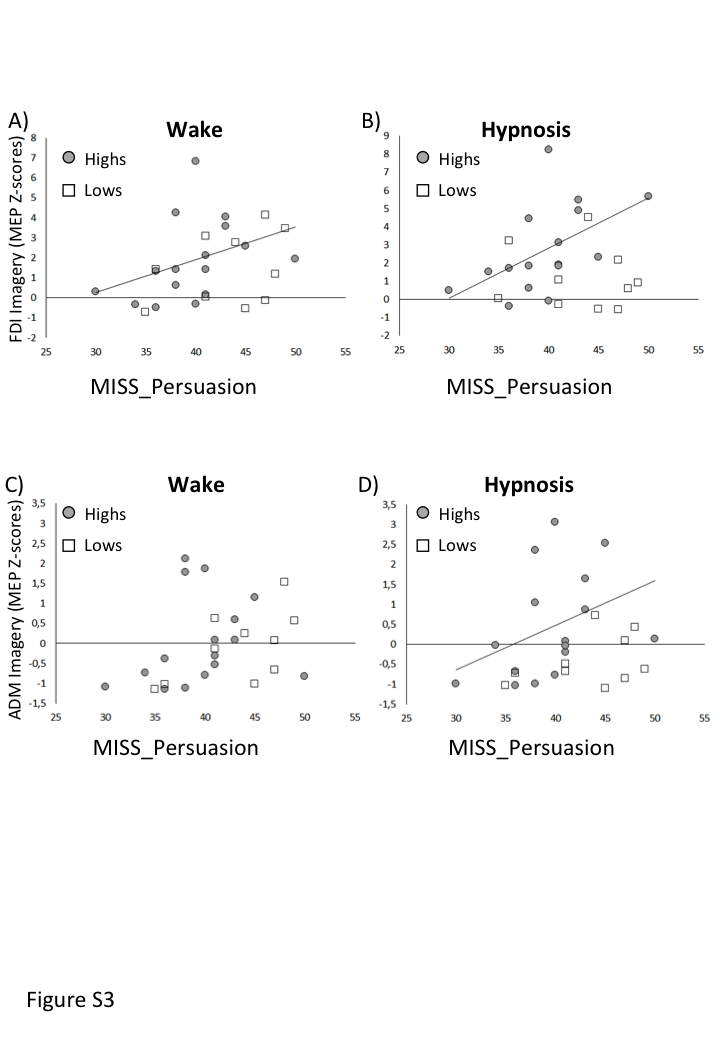

Supplement: Supplementary file 4 — Supplementary Figure S3. [file 41598_2020_74020_MOESM4_ESM.tiff]

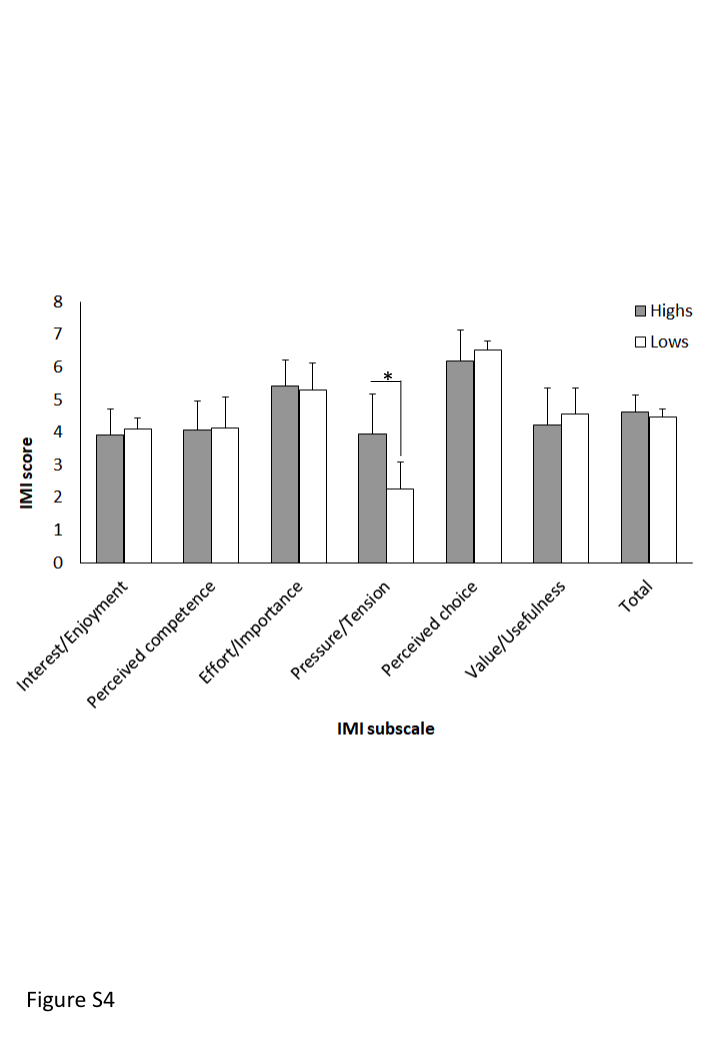

Supplement: Supplementary file 5 — Supplementary Figure S4. [file 41598_2020_74020_MOESM5_ESM.tiff]
